# A qualitative study on why did the poorly-educated Chinese elderly fail to attend nurse-led case manager clinic and how to facilitate their attendance

**DOI:** 10.1186/s12939-015-0137-3

**Published:** 2015-01-31

**Authors:** Susanna Lok Lam Hung, Sau Nga Fu, Po Shan Lau, Samuel Yeung Shan Wong

**Affiliations:** Department of Family Medicine and Primary Health Care, Kowloon West Cluster, Hospital Authority, 1/F, Our Lady of Maryknoll Hospital, Wong Tai Sin, Kowloon, Hong Kong S.A.R., China; Division of Family Medicine and Primary Healthcare, The Jockey Club School of Public Health and Primary Care, The Chinese University of Hong Kong, 4/F, School of Public Health, Prince of Wales Hospital, Shatin, N.T., Hong Kong S.A.R., China

**Keywords:** Nurse led clinic, Primary health care, Diabetes mellitus type 2, Patient dropout, Aged

## Abstract

**Objectives:**

This study explored the views, barriers and facilitators of the poorly-educated elderly who were non-attendee of the nurse-led case manager clinic. The case managers provide assessment for diabetes complication screening and can refer patients to the appropriate multidisciplinary team in public outpatient primary care setting.

**Methods:**

We adopted qualitative research method by individual semi-structured face to face interviews. Nineteen Chinese type 2 diabetes mellitus subjects aged ≥ 60 who failed to attend the nurse-led case manager clinic were interviewed. They all came from a socially deprived urban district in Hong Kong. Content and thematic analysis was performed.

**Results:**

Seven men and twelve women aged 60 to 89 were interviewed. Nine of them received no formal education and ten of them attended up to primary school. The reasons for non-attendance included attitude and poor knowledge towards diabetes complication screening and confusion of the nurse-led clinic as an educational talk. Most respondents could not understand the reason for the screening of diabetic complications, the concept of multidisciplinary care and the procedure and outcomes of nurse assessment. Five respondents were unable to follow multiple appointments because they could not read. Other reasons included physical barriers and comorbidity, family and financial constraint. They either had a tight daily schedule because of the need to take care of family members, or the family members who brought them to clinic had difficulty in attending multiple appointments. Enhanced understanding of the importance and procedure of diabetes multidisciplinary management, a flexible appointment system and a single clear appointment sheet may facilitate their attendance.

**Conclusion:**

Poorly-educated Chinese elderly with DM and their care givers faced physical, social and psychological barriers when attending the nurse-led case manager clinic. Strategies targeting on their low literacy include effective communication and education by health care professionals to arrive a shared understanding of care plan as well as a flexible appointment and schedule system.

## Background

Nearly 1 in 10 Chinese adults have diabetes [1]. The prevalence is estimated to range from 2% in people less than 35 years old to over 20% in those older than 65 [1,2]. In 2004, the cost of Type 2 diabetes contributed up to 3.4% of the total Hong Kong healthcare expenditure and 6.4% of the Hong Kong Hospital Authority’s (HKHA) expenditures on health [3]. There are approximately 190,000 patients receiving care for DM in the general outpatient clinics (GOPCs) according to data from HKHA [4].

A well-structured, comprehensive, multilayered and multifaceted approach to patients with chronic disease was shown to be able to improve patients’ clinical outcome [5]. The annual review of DM patients was recommended for universal use in the European St Vincent Declaration in 1990 [6]. Different kinds of structured primary care programs for type 2 diabetes mellitus (DM) patients that targeted at improving cardiovascular risk factors as well as glycemic monitoring and control have been launched in United Kingdom, [7] Australia [8] and New Zealand [9]. Previous studies have shown that the addition of a nurse who plays the role of providing patients with education interventions can lead to improvements in patient outcomes as well as the process of care [10,11]. Since August 2009, the public GOPCs in Hong Kong introduced an assessment and interventional multidisciplinary DM care program in primary care setting [4]. Nurses were trained to be case managers. They annually assessed patients’ cardiovascular risk factors and monitored the conduct of complication screening including retinopathy assessment, assessment of the presence of microalbuminuria, peripheral vascular disease and neuropathy. All data was recorded on the Computer Management System. The nurses also provided interventions including the education of patients on proper drug use, self-blood glucose monitoring and the management of hyperglycemia and hypoglycemia. They could also refer patients to dietitians, physiotherapist, mental health service, podiatrist, occupational therapist and ophthalmologist according to a standardized management protocol. The program was shown to improve glycemic control and reduce cardiovascular risk for the participants at 12 months follow-up [12]. The multidisciplinary approach seemed to be particularly important for the elderly due to their elevated risk for diabetes complications and other comorbidities such as depression, cognitive impairment, chronic pain, visual impairment and polypharmacy [13]. Despite positive results from these programs, there were reports of non-attendance to various diabetes clinics [14-16]. Moreover, patients who failed to attend these diabetes clinic tended to have significantly more risk factors and complications than those who keep their appointment [17].

Reasons of failure to attend DM clinics identified in previous studies include affordability, accessibility, [18] efficiency of care and awareness of support resources [18,19]. Other influential factors include patient’s co-morbidities, psychosocial problems, family role [16] and lack of social support [19-22]. Patients’ health beliefs, attitudes, culture and literacy level also affect their diabetes self-management [19]. Patients with low income, [23] low educational level and low literacy [24] were found to experience significant barriers to health care as well as a lower treatment satisfaction [25] and a poorer health outcome [26]. Yet they were more likely to be non-attenders in general practice [27]. It is therefore pressing to know what are views and understanding of the poorly educated elderly DM patients to their disease, its complications and the multidisciplinary care provided to them, as well as the factors which contribute to their failure to attend the important case manager clinic under their particular social context.

Several Western studies suggested strategies to reduce non-attendance in general practice. Advanced access scheduling, [28] reminder system, [29] increase motivation and orientation statement [27] may improve attendance. The question arises as to whether the suggested strategies equally apply to poorly-educated Chinese elderly with diabetes in Primary Care setting.

### Objective

We aimed to explore the views on diabetes complication screening clinic, the reasons for non-attendance and strategies to facilitate the attendance of diabetes clinics among poorly-educated elderly Chinese who failed to attend clinic appointments.

## Methods

### Study design

This qualitative study adopted individual face to face interviews. Written informed consent ensuring anonymity and confidentiality were obtained. Ethics approval was obtained from the local Hospital Authority Kowloon West Cluster Research Ethics Committee for the study protocol.

### Sample frame

This study adopted a purposive sampling method. Subjects were recruited from 3 study primary care outpatient clinics situated in Wong Tai Sin district of Hong Kong. It is an area that is densely-populated with public housing estates and has the highest proportion of population aged > 60 (23.1%) compared to 19.1% in the entire population in Hong Kong [30]. Their household income was also ranked the second lowest among total 19 districts [31]. Research subjects recruited were likely to be representing the socially disadvantaged elderly. There were an estimated total of 6,200 diabetes patients regularly attending the 3 study clinics in 2010. Local statistics showed there were 831 patients (25.8% of total booked appointment) who failed to attend the nurse clinic in 2010. Five Hundred and eighteen (62.3%) of them were aged 60 or above.

### Inclusion and exclusion criteria

A list of subjects aged ≥ 60, who failed to attend the nurse-led complication screening clinic from Nov 2011 to May 2012 was obtained from the computer system. Chinese subjects who fulfilled DM diagnostic criteria [32] and were able to consent and communicate, were recruited via brief telephone introduction. Subjects who had subsequently rebooked nurse-led clinic or had active or unstable psychiatric illness were excluded.

### Process of interviews

Individual face to face interviews were conducted in Cantonese by one of the authors (SH, SF and PL) in consultation rooms of the study clinics. We avoided interviewing our own patients in order to minimize bias and stress to respondents. We followed the semi-structured interview guideline (Appendix 1) that were constructed after relevant literature review. The interview process was recorded to audio files.

### Translation and transcription

The interview audio files were transcribed verbatim into Chinese by research technicians. Authors continued to listen to the recordings and check for accuracy of all transcripts. Selected texts were translated to English by two of the authors independently. Final version of selected text were agreed upon regular meeting.

### Data analysis

The transcripts were analyzed manually and supplemented by computer software NVivo® using iterative/ thematic analysis and the grounded theory approach [33]. A coding tree with clear definition and operation guideline for each code was formulated by the first and second authors. The first and second authors then performed inter-coding of the verbatim transcription independently in line with the coding tree. Two meetings were held where all authors exchanged their views and ensured consistency and quality of the coding data process. The first and second author performed content analysis, textual analysis and narrative analysis for singling out the important patterns, authentic features, manifest and latent meaning as well as remarkable issues and stories from the interview findings. We continued the recruitment of participants until data saturation was achieved with no new themes emerged from the qualitative interviews.

## Results

### Socio-demographic characteristics

The characteristics of the 19 respondents were shown in Table 1. The socio-demographic statistics were summarized in Table 2. The age of respondents ranged from 60–87 years with an average of 74 years of age (S.D. 9.0). Thirteen of them suffered from some kind of established diabetic complications (e.g. nephropathy, retinopathy, peripheral vasculopathy) and 17 also suffered from other co-morbidities. The control of diabetes was optimal as revealed by the mean HbA1c level of 6.8%. All respondents were either retirees or housewives, and had primary level education or below.

**Table 1:** **Sociodemographic and clinic characteristic of 19 respondents**

**Respondent code**	**Age**	**Sex**	**Duration of diabetes (years)**	**Latest HbA1c**	Co-morbidities*	**Diabetes complications**	**Occupation**	**Social allowance**	**Education**
P1	78	F	6	6.6%	HT	MA, renal impairment	Retired	No	Primary
P2	83	F	15	5.4%	HT, atrial fibrillation, heart failure	MA	Retired	No	Nil
P3	82	M	13	6.1%	HT, knee osteoarthritis, poor vision	MA, renal impairment, glaucoma	Housework	No	Nil
P4	65	F	1	7.1%	HT, stroke	MA, DR	Housewife	No	Primary
P5	79	M	13	7.9%	Knee osteoarthritis	Renal impairment, leg ulcer	Retired	Yes	Primary
P6	83	F	7	6.5%	HT, knee osteoarthritis, low back pain	Nil	Retired	Yes	Nil
P7	77	F	22	7.1%	HT, knee osteoarthritis	Nil	Retired	No	Nil
P8	80	M	14	5.4%	Nil	MA, DR	Retired	No	Primary
P9	61	F	7	6.2%	Nil	Nil	Unemployed	No	Nil
P10	62	M	1	5.6%	HT	Nil	Manual worker	No	Primary
P11	60	M	6	7.1%	HT	Nil	Retired	No	Nil
P12	65	M	10	6.3%	HT, chronic obstructive airway disease	MA	Retired	Yes	Primary
P13	87	F	11	6.3%	IHD, heart failure, stroke, hip fracture	MA, DR, IHD	Retired	No	Nil
P14	72	F	16	6.9%	HT	MA	Housewife	No	Primary
P15	84	F	17	8.0%	HT	Peripheral vascular disease	Housewife	No	Primary
P16	78	F	14	7.4%	HT	DR	Housewife	No	Nil
P17	79	F	5	7.1%	HT	Nil	Housewife	No	Nil
P18	73	M	8	6.9%	HT	Renal impairment	Retired	No	Primary
P19	60	F	7	9.1%	Nil	DR	Housewife	No	Primary

*HT = Hypertension, IHD = Ischaemic heart disease, MA = Microalbuminuria, DR = Diabetic retinopathy.

**Table 2:** **Summary of sociodemographic and clinical characteristics of respondents**

**Characteristics (n, %)**			
*Socio-demographic*			
	Gender		
		Female	12
		Male	7
	Age (year, mean ± SD)		73.8 ± 8.97
	Occupation		
		Employee	1
		Retired	10
		Housewife	7
		Umemployed	1
	Education		
		Nil	9
		Primary	10
		Secondary	1
* Clinical*			
	Duration of DM (year, mean ± SD)		9.85 ± 5.58
	Latest HbA1c level		6.8% ± 0.9%
	Diabetes Complication		
		Retinopathy	5
		Microalbuminuria	8
		Renal impairment	4
		Stroke	2
		Cardiovascular disease (CVD)	2
		Eye complication	2
		Leg ulcer	1
		Peripheral Vascular disease	1
	Comorbidity		
		Hypertension	15
		Musculoskeletal problem	6
		Other Heart Disease (exclude CVD)	3
		Chronic obstructive airway disease	1

### Reasons for not attending the nurse-led diabetes complication screening clinic

We identified four main themes that accounted for respondents’ non-attendance.

### Theme 1: negative attitude and poor knowledge towards diabetes, diabetes complication, complication screening procedure and the subsequent management

#### Preventive screening is not important/useful/urgent

Many subjects felt they were asymptomatic and hence preventive screening for complications was not necessary. They felt that the screening examination performed was repetitive and recalled being told the same findings every year. There were no subjective improvements resulted from the screening.

Mrs. P9 (61-year-old):

“I found it useless. The nurse seemed to be only having a brief look and just tapping (my leg with a tuning fork). They told me that my eyes were normal… a normal report means that I am normal”.

#### Not everyone needs to attend preventive screening

Some participants perceived complication screening is only needed for patients with poor diabetes control. But their views on assessing the severity of diabetes were variable and sometimes vague. Some based their judgment on home glucose monitoring, presence of leg discomfort, vision impairment, mobility problem and the quality of sleep and appetite.

#### Confusion of the clinic as education talk

Some participants misunderstood that the diabetes complication screening clinic is an educational talk instead of a one-to-one nurse assessment clinic.

#### Fear the side effect of pupil dilation during retinal photo

Two respondents feared that the transient blurring and discomfort resulted from pupil dilatation would damage their eyes. They felt the eye examination was time consuming and the need for relative’s accompany was troublesome.

Mr. P8 (80-year-old)

“I was told I needed to be accompanied by a family member (to attend the retinal photo examination) but they all need to work… I fear this (pupil dilatation) would damage my eyes…” He felt disappointed because no eye drop was prescribed after the procedure to relieve his discomfort.

Mr. P12 (65-year-old) expressed emotional stress and fear when recalling the retinal photo examination. He needed to attend the same examination for a second time after being told that the first examination was unsuccessful. Apart from physical discomfort it had caused him anxiety as he lived by himself.

### Theme 2: difficulty in following multiple appointments

The respondents expressed difficulty in reading appointment slips particularly when given with multiple slips. Some developed their own coping strategies. However, mistakes still happened such as reading the wrong dates or forgetting the meaning of the symbols as marked on the calendar.

#### Low literacy

Five of the respondents (Mrs P4, 65-year-old; Mrs. P9, 61-year-old; Mr. P11, 60-year-old; Mrs P16, 78-year-old; Mr. P18, 73-year-old) revealed that their illiteracy imposed a significant barrier to keep track of clinic appointments. Some overcome this barrier by relying on family members or neighbors to read and remind them of appointment dates.

Mr. P11 (60-year-old): “I don’t know I have to come back because I can’t read”.

Mrs. P16 (78-year-old) developed other strategies because she lived alone: “I can’t read (laughing embarrassingly), and I’m also forgetful… so I mark a circle in the daily calendar. I check the calendar every day. When I see a circle I have to think hard about what the event is supposed to be for that day…”.

#### Not aware of the clinic appointment booking

As described earlier on, the diabetes complication screening nurse clinic is only part of the annual diabetes assessment. Patients often need multiple clinic visits to complete the full assessment.

Mr. P5 (79-year-old) was confused by the multiple appointment slips. “I was given multiple appointment slips at one time, with different dates for blood taking and many others… eventually I only missed one of the appointments (the nurse clinic)”.

Mrs. P19 (60-year-old) lost the appointment slip. “I don’t know when to come back… But if I’ve got that paper, I will remember…”.

The problem was further complicated when Mr. P18 (73-year-old) needed to attend various specialty clinics in different hospitals. “I’ve got headache when faced with so many appointments”.

### *Theme 3:* physical barriers and comorbidities

Most respondents expressed forgetting the appointment simply due to declined memory or by mistake. Others suffered mobility problems such as flaring up of back and lower limb pain that made them physically difficult to attend the clinic.

Due to suffering from dementia, P18 (73-year-old) was fully dependent on her daughter and domestic helper for activities of daily living including transport to clinic for any procedures or consultations. She could not attend clinic when her daughter could not take leave from work.

### *Theme 4:* family and financial constraint

#### Time restraint due to family commitments

Mrs. P7 (77-year-old) was not only the caregivers for the family but also needed to look after her grandchildren. She could not attend the nurse-led clinic because she needed to deliver lunch to and pick up grandchildren from school.

#### High transport costs

All respondents felt the charge for nurse clinic (USD6) was reasonable and affordable. However the financial burden of high transport costs far outweighed the clinic charge. Mr. P18 (73-year-old)’s daughter explained, “The taxi fee was unpredictable. Hence, I will drop out some of the “less important” appointments for my father”.

### How to facilitate Chinese elderly attendance

Eighteen out of the nineteen respondents declared that they would try their best to attend if doctors and nurses clearly explained the importance and when to attend.

Some respondents requested a more flexible appointment system. Mrs P7 suggested spacing out all clinic visits to fit her schedule because she needed to take care of her grandchildren. In contrary, Mr. P18’s daughter requested to complete the entire complication assessment on the same day to minimize taking leave from work.

Both Mrs. P13 (87-year-old) and Mrs P15 (84-year-old) suggested that providing a single, clear instruction sheet for all appointments rather than multiple appointment slips would help them comprehend and remember.

## Discussion

While most previous studies on the reason of non-attendance in primary care focused on general diabetic subjects and all types of clinic visits related to diabetes, [16,23,34] this is the first report focusing on the views of (exclusively) poorly-educated elderly Chinese on nurse-led diabetes complication clinic. Attendance to the clinic is one of the access points to a multidisciplinary diabetes care program, as these programs are recommended in most updated international guidelines [35-37]. The respondents resided in a socially deprived urban area of Hong Kong.

The respondents perceived low importance of the complication screening service because they were asymptomatic from diabetes or had good diabetes control. This observation is supported by findings from other studies [21,38]. Such perception may be explained by their low level of medical knowledge, as shown by the fact that they could hardly understand what the nurses did during the complication screening clinics. They also had no or wrong ideas about the retinal photo screening procedure and vague understanding of diabetes complications.

In line with findings by Griffin, the confusion or misconception about the role of diabetes clinic contributed to the problem of poor attendance [15]. Another finding in our study was that participants were fearful of potential eye damage from taking retinal photo and the need for relative’s company for such examination. These individual barriers to diabetic retinopathy screening were also reported by a recent study [39].

Patients with low education level were found to have an increased risk of non-attendance to diabetes care [27]. The underlying reasons might be as described by our respondents such as encountering difficulties with reading the appointment booking slips as well as remembering the different appointment dates. The coping strategies described were very similar to the finding of a study conducted in Hong Kong for low literacy patients [24].

Our study found that respondents with multiple chronic conditions (multi-morbidity) were faced with significant barriers to self-management due to the simultaneous demands of competing morbidities such as back pain, fatigue, arthritis, chronic lung or heart problems [18,19,40] as suggested in other earlier studies. It was observed that lack of social support, financial pressure and time restraint due to family commitments affected participants’ attendance. About 35% of our participants were either partially or fully dependent on their family or caregivers for diabetes care due to old age, poor mobility or lack of adequate self-care. The observation that living with diabetes challenged established family roles and that health is a family responsibility is consistent with findings in previous studies [41,42]. In our study, it was noted that whether or not to attend a complication screening may not be entirely a patient’s decision and may even create family conflict. Chesla et al. reported sometimes beliefs varied about who in the family should be responsible for managing the disease create conflicts in negotiating differing role expectations [43].

On the other hand, the phenomenon of being preoccupied with family responsibilities such the responsibility to look after one’s grandchildren was described by previous studies on immigrant Chinese Americans [34,42]. Grandparents traditionally assume active family roles in Chinese culture and they have little time to properly care for their diabetes [34]. In a study by Alberti et al., patients and health care workers described travel cost as one of the financial barriers influencing various aspects of diabetes care [23]. This is similar to the finding in our study.

In order to facilitate these patients to understand and to attend diabetes complication screening, clearer communication at patients’ level and shared decision making with health care professionals [43] would be important. Previous study suggested that a diabetes disease management program that addressed literacy was able to improve health outcomes and might be particularly beneficial for patients with low literacy [44]. Health care professionals should acknowledge the patients’ competing family and social needs when arranging appointments for patients. A more flexible scheduling and appointment system can help to facilitate elderly’s attendance by minimizing the financial cost and tailoring the appointment time to the individual’s need.

The fact that many participants commented that getting a drug prescription for their diabetes is of utmost importance is a concern. Many did not understand the rationale for a comprehensive diabetes care plan. This is not consistent with the principles of an effective diabetes management where a multidisciplinary approach is widely being adopted.

### Study limitation

We might not able to obtain impartial views from respondents because the investigators who interviewed these patients were physicians (although not the ones who look after these patients). Respondents might not disclose their negative feelings and thoughts. The low education level of respondents might not be fully ableto express their views and suggestion about the service provided.

## Conclusions

In the primary care setting, poorly-educated Chinese diabetes elderly failed to attend the nurse-led diabetes complication screening clinic because of their negative attitude and poor knowledge towards diabetes complication screening, physical barriers and comorbidity, social and financial constraints and difficulty in following multiple appointments due to low literacy. Clear and shared understanding of the importance and procedure of diabetes complication screening, a flexible appointment system and a single clearly written appointment sheet may facilitate their attendance.

## Appendix 1: Semi-structured interview guide

- ❖ *These questions are a guide to help the interviewers to remember topics they want to include. Interviewers are also free to ask additional questions during the interview and to respond to issues or questions raised by the participant. (1)*
- ❖ *The key questions are presented by thematic category here but the order of presentation may differ in individual interviews depending on the flow of the interview and participants’ response.*
- ❖ *Interviewers need to build rapport, listen carefully and allow pauses and thinking time for the participant. (1)*
- ❖ *For simplicity, the word “clinic” refers to “diabetes complication screening nurse clinic” in this guide.*
- ❖ *Questions marked with “*” were used in previous studies to attempt to determine beliefs about health and illness related to diabetes. (2)*

### I. Introduction

- Welcome
- Present to the participants the idea of semi-structured interviews
- Explain the topic of interview and why their views are important

### II. Start with a broad question

Many patients encounter problems/barriers in attending our clinic. For example, it could be related to understanding about the clinic, health belief, cultural belief, relationship with healthcare professional, structural or financial barriers, etc … We understand that you did not attend our clinic as scheduled. We are interested to find out why. Can you tell me about your views and experience?

- ❖ *Allow elaboration*
- ❖ *Probe by asking for clarification, details and examples: e.g. “How so?”, “Could you elaborate?”, “Tell me more about…” (1)*

### III. Focus on specific themes if not covered

#### *1) Knowledge of diabetes and diabetes clinic*

- What do you think has caused your diabetes? (*)
- Why do you think it started when it did? (*)
- What do you think your diabetes does to you? (*)
- How severe is your diabetes? Will it have a short or long term course? (*)
- What kind of treatments do you think you should receive? (*)
- What are the chief problems your diabetes has caused for you? (*)
- What are the most important results you hope to receive from attending treatment? (*)
- Could you share your understanding about the role of complication screening clinic in your diabetes care?
- How important is it to your diabetes management?
- Do you think there is a need to attend the clinic?

#### *2) Personal health belief*

- What do you think of Western medicine when compared to traditional Chinese medicine/alternative medicine? For example, in terms of knowledge, understanding, familiarity, trust, accessibility/availability… etc
- If you have treated your diabetes with Chinese/alternative medicine in the past, can you tell me more about your beliefs and experience.
- Has this affected your decision to attending the clinic? If so, in what way?
- Do you think there is a gap between what you know you should do about your diabetes and what you are actually doing?

#### *3) Cultural belief*

- Does looking after your diabetes come into conflict with your culture or belief? If so, could you describe specifically challenging or difficult situations you have come across?
- Would you mind telling me who do you live with? What is your role in your family (e.g. breadwinner, household chores, carer for elderly or young children)? Does attending the clinic cause significant disruption to your daily life? If so, in what way? How do you cope?
- Do your family members help you with looking after your diabetes? Also, are they involved in healthcare decisions, including decision to attend clinics? Has there ever been any conflict or disagreement about healthcare decisions? If so, can you tell me more about it?

#### *4) Emotional/psychological barrier*

- Do you ever suffer psychological or emotional stress with diabetes? If so, can you describe in what way does it affect you in attending our clinic?
- What do you fear most about diabetes? (*) How do you cope with it? (e.g. denial, ignorance, discuss with other people, seek alternative treatment)

#### *5) Interaction with physicians or health care workers*

- How did your doctor explain to you about why do you need to attend the complication screening? How well did the doctor help you understand all the information? Did the doctor involve you when deciding to book the clinic? Did you and the doctor select the treatment option together? (3)

If you have attended the clinic before:

- Describe your experience.
- How useful was the nurse clinic to you?
- Do you perceive any improvement after attending the clinic?
- How would you improve these experiences, if at all?

#### *6) Structural and financial barrier*

- Forget about appointment
- Appointment time
- Transport
- Other priorities/Social issues
- Other disability e.g. mobility, self-care, higher functioning
- Financial cost

If you needed to pay for the clinic service:

- Was the cost an issue for you?
- Does your financial status affect your attendance to our clinic?

### III. Finding a solution

- Of the topics discussed so far, what do you consider to be the most important barrier to attending the clinic?
- Have you tried to overcome the difficulties/barriers as mentioned earlier? If so, what did you do?
- Of the barriers/problems discussed so far, are there any ideas on areas we can improve to help you attend the clinic?

### IV. Conclusion

- Wrap up by interviewer of the issues discussed and clarify with participant “Do you think this is an adequate summary?”
- Is there anything you would like to share with us that we did not ask you or you did not get to chance to?
- Thank participant for their time and contribution
